# Triptolide Modulates the Expression of Inflammation-Associated lncRNA-PACER and lincRNA-p21 in *Mycobacterium tuberculosis*–Infected Monocyte-Derived Macrophages

**DOI:** 10.3389/fphar.2021.618462

**Published:** 2021-04-12

**Authors:** Ousman Tamgue, Julius Ebua Chia, Frank Brombacher

**Affiliations:** ^1^ Department of Biochemistry, Faculty of Sciences, University of Douala, Douala, Cameroon; ^2^ International Centre for Genetic Engineering and Biotechnology (ICGEB), Cape Town Component, Cape Town, South Africa; ^3^ Department of Pathology, Faculty of Health Sciences, Institute of Infectious Diseases and Molecular Medicine (IDM), Division of Immunology and South African Medical Research Council (SAMRC) Immunology of Infectious Diseases, University of Cape Town, Cape Town, South Africa; ^4^ Wellcome Centre for Infectious Diseases Research in Africa, Institute of Infectious Diseases and Molecular Medicine (IDM), Faculty of Health Sciences, University of Cape Town, Cape Town, South Africa

**Keywords:** long non-coding RNAs, triptolide, Mycobacterium tuberculosis, macrophages, inflammation

## Abstract

Triptolide is a diterpene triepoxide, which performs its biological activities *via* mechanisms including induction of apoptosis, targeting of pro-inflammatory cytokines, and reshaping of the epigenetic landscape of target cells. However, the targeting of long non-coding RNAs (lncRNAs) by triptolide has not yet been investigated, despite their emerging roles as key epigenetic regulators of inflammation and immune cell function during *Mycobacterium tuberculosis* (Mtb) infection. Hence, we investigated whether triptolide targets inflammation-associated lncRNA-PACER and lincRNA-p21 and how this targeting associates with Mtb killing within monocyte-derived macrophages (MDMs).Using RT-qPCR, we found that triptolide induced the expression of lincRNA-p21 but inhibited the expression of lncRNA-PACER in resting MDMs in a dose- and time-dependent manner. Moreover, Mtb infection induced the expression of lincRNA-p21 and lncRNA-PACER, and exposure to triptolide before or after Mtb infection led to further increase of Mtb-induced expression of these lncRNAs in MDMs. We further found that contrary to lncRNA-PACER, triptolide time- and dose-dependently upregulated Ptgs-2, which is a proximal gene regulated by lncRNA-PACER. Also, low-concentration triptolide inhibited the expression of cytokine IL-6, a known target of lincRNA-p21. Mtb infection induced the expression of IL-6 and Ptgs-2, and triptolide treatment further increased IL-6 but decreased Ptgs-2 expression in Mtb-infected MDMs. The inverse relation between the expression of these lncRNAs and their target genes is concordant with the conception that these lncRNAs mediate, at least partially, the cytotoxic and/or anti-inflammatory activities of triptolide in both resting and activated MDMs. Using the CFU count method, we found that triptolide decreased the intracellular growth of Mtb HN878. The alamarBlue assay showed that this decreased Mtb HN878 growth was not as a result of direct targeting of Mtb HN878 by triptolide, but rather evoking MDMs’ intracellular killing mechanisms which we speculate could include triptolide-induced enhancement of MDMs’ effector killing functions mediated by lncRNA-PACER and lincRNA-p21. Altogether, these results provide proof of the modulation of lncRNA-PACER and lincRNA-p21 expression by triptolide, and a possible link between these lncRNAs, the enhancement of MDMs’ effector killing functions and the intracellular Mtb-killing activities of triptolide. These findings prompt for further investigation of the precise contribution of these lncRNAs to triptolide-induced activities in MDMs.

## Introduction

Triptolide is one of the active compounds of the medicinal herb *Tripterygium wilfordii Hook f*., which has been widely used in China to treat various conditions and diseases including rheumatoid arthritis, nephritic syndrome, lupus, and Behcet’s disease. This diterpene triepoxide possesses antitumor, immune-suppressive, and anti-inflammatory activities which it exerts *via* several mechanisms in a tissue-, context-, and disease-specific manner (Zhao et al., 2010a; Zhao et al., 2010b; Liu, 2011; Carter et al., 2012; Tamgue et al., 2013; Wu et al., 2013; Hou et al., 2017; Tamgue and Lei, 2017; Chen et al., 2018; Yang et al., 2018; Huang et al., 2019; Yuan et al., 2019; Zhao et al., 2019). Notably, triptolide exerts its cytotoxic activities through induction of DNA damage, cell cycle arrest, apoptosis, and autophagy in several cell types (Park and Kim, 2013; Zhang et al., 2016; You et al., 2018). Its anti-inflammatory activities result among other mechanisms from the downregulation of NF-kb– and AP-1–controlled pro-inflammatory molecules such as TNF-α, IL-6, IL-12, and Ptgs-2 in several cell types, including macrophages and dendritic cells (Chun and Surh, 2004; De Moraes et al., 2007; Liu et al., 2007; Lu et al., 2014; Wang et al., 2014; Chen et al., 2018).

Indeed, prostaglandin synthase-2 (Ptgs-2) also known as cyclooxygenase-2 (Cox-2) gene is the inducible rate-limiting enzyme in the biosynthesis of the prostanoid PGE2, a lipid mediator which possesses both pro- and anti-inflammatory activities (Tilley et al., 2001), and contributes to both the promotion and inhibition of programmed cell death (Zamora et al., 1998; Chen et al., 2008). Interleukin-6 (IL-6) is a soluble mediator which is produced immediately and transiently in response to tissue injury and infection (Zhang et al., 1994; Tanaka et al., 2014). This cytokine has pleiotropic effects on hematopoiesis, immune response, and inflammation (Tanaka et al., 2014). It has been assigned with controversial roles in inflammation and cell faith which seem to be context- and tissue-dependent (Mauer et al., 2015; Linnemann et al., 2017). Ptgs-2 and IL-6 expressions are regulated through several mechanisms involving NF-IL6, AP-1, and NF-kB signaling pathways (Zhang et al., 1994; Liu et al., 2007; Tanaka et al., 2014). Recently, the discovery of long noncoding RNAs (lncRNAs) has added another layer of complexity on the regulation of these inflammatory genes expression and activities.

Long noncoding RNAs are a subtype of non-protein coding RNA species (at least 200 nucleotides in length), which have emerged as key regulators of the development and function of immune cells such as macrophages, dendritic cells, and T lymphocytes. They perform their activities through cis-regulation of nearby proximal genes and trans-regulation of distant genes located on other chromosomes (Heward and Lindsay, 2014; Chen et al., 2017; Flores-Concha and Onate, 2020). Such lncRNAs include lincRNA-p21 and lncRNA p50-associated COX-2 extragenic RNA (PACER). lincRNA-p21 is a p53-induced lncRNA which plays important roles in inflammation and cell response to DNA damage. With regards to inflammation, lincRNA-p21 mediates the anti-inflammatory activities of the transcription factor p53 (Komarova et al., 2005; Huarte et al., 2010). However, lincRNA-p21 was reported to display both anti-inflammatory and pro-inflammatory roles depending on the stimulators, cell types, or specific disease context (Spurlock et al., 2014; Yang et al., 2014; Zhou et al., 2016; Ye et al., 2018a). For instance, lincRNA-p21 induces the expression of the pro-inflammatory cytokine IL-6 in LPS-stimulated BV2 microglia cells (Ye et al., 2018a). As for the involvement in cellular response to stress and DNA damage, lincRNA-p21 expression was induced in several cells and tissues exposed to genotoxic agents (Recio et al., 2013; Gezer et al., 2014; Tang et al., 2015). Here, the mechanisms of lincRNA-p21 action include positive transcriptional regulation of its neighboring gene p21, and negative regulation of distant genes involved in the p53 transcriptional network, resulting in the induction of p53-dependent cell cycle arrest and apoptosis in numerous cell types (Huarte et al., 2010; Dimitrova et al., 2014; Tu et al., 2017; Jin et al., 2019). lncRNA-PACER (also known as lncRNA-Cox-2) is induced in pro-inflammatory conditions, including in IL-1b–stimulated chondrocytes (Pearson et al., 2016) and LPS-stimulated monocytes (Krawczyk and Emerson, 2014). Mechanistically, lncRNA-PACER is a positive regulator of its proximal pro-inflammatory gene Ptgs-2 *via* mechanisms involving the sequestration of repressive NF-κB subunit p50 away from Ptgs-2 promoter; hence, it has been described as a pro-inflammatory lncRNA (Krawczyk and Emerson, 2014).

Host lncRNA’s expression is altered by *Mycobacterium tuberculosis* (Mtb), the causative agent of tuberculosis which is the first cause of mortality from a single infectious agent worldwide (Organization, 2019). Mtb hijacks host noncoding RNAs to evade deleterious host immune response including induction of pro-inflammatory cytokine and apoptosis (Wang et al., 2015; Huang et al., 2018; Roy et al., 2018; Tamgue et al., 2019). lincRNA-p21 is a positive regulator of apoptosis, a programmed cell death that contributes to host defense against intracellular pathogens (Lam et al., 2017). HIV-1 for instance targets lincRNA-p21 to inhibit apoptosis and ensure its survival within infected macrophages (Barichievy et al., 2018). lncRNA-PACER promotes the activation of macrophages towards the pro-inflammatory M1 phenotype known to be very efficient in killing Mtb (Ye et al., 2018b). IL-6 and Ptgs-2 pro-inflammatory genes are induced by mycobacterial LPS and LAM and play key role in cellular immune response against Mtb (Zhang et al., 1994; Nagabhushanam et al., 2003; Martinez et al., 2013; Tanaka et al., 2014; Jung et al., 2017; Xiong et al., 2018).

The World Health Organization has set the research and development of new drugs as one of the top priorities for the control and eradication of this deadly disease which is still being treated with drugs developed in the 60’s (Organization, 2019). Therefore, natural compounds like triptolide are good avenues to explore for the identification of new leads and the development of host-directed adjunctive therapies against TB. To our knowledge, there is only one report on lncRNAs as mediators of triptolide’s activities, and that report investigated the association between lncRNAs and triptolide-induced male mouse infertility (Xiong et al., 2019). Our work is, therefore, the first to investigate lncRNAs as mediators of triptolide’s activities on macrophages, which are key immune cells involved in the host immune response against Mtb. In this study, we provide first evidences that triptolide modulates the expression of lncRNA PACER and lincRNA-p21 in both resting and Mtb-infected human macrophages, which was associated with the modulation of their target genes IL-6 and Ptgs-2. This work conjectured a possible link between these lncRNAs expression, the specific enhancement of pro-inflammatory activities in macrophages, and the intracellular Mtb-killing activities of triptolide. These results prompt for lincRNA-p21 and/or lncRNA-PACER gain-/loss-of-function experiments, which will unequivocally identify their targets in MDMs and highlight their precise contribution to triptolide’s biological activities on both resting and Mtb-infected human macrophages.

## Materials and Methods

### Ethics Statement

The recruitment of healthy volunteers for this study was approved by the Human Ethics Committee, Faculty of Health Sciences, University of Cape Town, Cape Town (HREC Ref Number: 635/2015). Inclusion criteria were as follows: age 18–50 years, both sex, no history of TB, no contact with TB patients, HIV negative, sputum smear negative, non-smokers, no chronic alcoholism, normal chest x-rays, no chronic disease, not receiving immunosuppressive therapy, IGRA-negative, and absence of other pulmonary diseases. The participants who did not meet the above criteria, did not consent to signing the inform consent form or to undertaking an HIV test were excluded from this study.

### Generation of Monocyte-Derived Macrophages

PBMCs were isolated as described previously (Tamgue et al., 2019). For the generation of MDMs, PBMCs were plated in 12- or 96-well tissue culture plates (Corning Costar®, Cambridge, MA) at a density of 15 × 10^6^ or 1,5 × 10^6^ cells per well, respectively, and incubated (37 °C; 5% CO_2_; and 70% RH) for 2 h to allow monocytes to adhere. Non-adherent cells were discarded and adherent monocytes were given a gentle wash with PBS and then incubated in X-VIVO™ 15 serum-free hematopoietic medium (supplemented with 1% penicillin G/streptomycin) for 7 days to allow for the differentiation of monocytes into MDMs. X-VIVO™ 15 serum-free hematopoietic medium was changed on day 4. On day 7, X-VIVO™ 15 was removed, MDMs were washed once with PBS and complete growth medium (RPMI 1640 medium supplemented with 10% FCS, 2 mm l-glutamine, and 1% penicillin G/streptomycin. All purchased from Life Technologies™, Carlsbad, CA, United States) added for downstream experiments. MDMs purity was assessed by fluorescence-activated cell sorting (FACS) analysis using a PE–labeled anti-CD11b, PerCP-labeled anti–HLA-DR, FITC-labeled anti-CD14, and APC-labeled anti-CD3 monoclonal antibodies (All purchased from BD Biosciences™ CA, United States). MDMs purity was more than 95%.

### Triptolide Treatment and *Mycobacterium tuberculosis* Infection of Monocyte-Derived Macrophages

For concentration dependence experiments, 1,5 × 10^6^ MDMs were plated in a 12-well tissue culture plate, then treated with low-concentration (1, 10 nM) and high-concentration (100 nM) triptolide (T3652-1 MG, > 98% pure, Sigma-Aldrich/Merck, Germany) for 6 h (short time) and 24 h (long time). Same volume of DMSO was used as vector control. In all experiments, DMSO was added to the culture medium at a final concentration of 0.01% v/v. For time-dependence experiments, MDMs were treated with 100 nM triptolide for 6, 12, and 24 h. For Mtb infection experiments, MDMs were treated with 1, 10, and 100 nM triptolide for 6 h before (pretreatment) or after (posttreatment) Mtb infection. MDMs were infected with Mtb strain HN878 (clinical hypervirulent strain) at a multiplicity of infection (MOI) of two bacilli: one cell (2:1). After 4 h, the supernatant was removed and fresh complete growth medium containing 10ug/ml gentamicin was added to remove extracellular bacteria. 2 h later, the medium was replaced by complete growth medium containing triptolide at the initial concentration and returned to the incubator for up to 24 h. Total RNA was then extracted at all time points indicated earlier.

### RNA Extraction

Cells were lysed in Qiazol (Qiagen, Germany) at different time points posttreatment and lysates were stored at −80 °C. Total RNA was isolated from the lysate using miRNeasy Mini Kit (Qiagen, Germany) according to the manufacturer’s instructions. RNA quantity and purity were measured using the ND-1000 NanoDrop spectrophotometer (ThermoScientific, DE, United States).

### cDNA Synthesis and Quantitative Real-Time PCR

100 ng total RNA was reverse transcribed into cDNA using Transcriptor First Strand cDNA Synthesis Kit (Roche, Germany) according to the manufacturer’s instructions. Quantitative real-time PCR (qPCR) was performed using LightCycler® 480 SYBR Green I Master (Roche, Germany) and gene-specific primers (IDT, CA, United States). Fold change in gene expression was calculated by the ΔΔCt method and normalized to Hprt1, which was used as internal control. The 0 h/DMSO-treated/non-infected samples were set to 1 (calibrator). The sequences of qPCR primer sets used were as follows: **hs-lncRNA-PACER**: forward 5′-TGT​AAA​TAG​TTA​ATG​TGA​GCT​CCA​CG-3′, reverse 5′-GCA​AAT​TCT​GGC​CAT​CGC-3′; **hs-lincRNA-p21**: forward 5′-GGG​TGG​CTC​ACT​CTT​CTG​GC-3′, reverse 5′-TGG​CCT​TGC​CCG​GGC​TTG​TC-3′; **hs-Hprt-1**: forward 5′-AGG​CGA​ACC​TCT​CGG​CTT​T-3′, reverse 5′-AAG​ACG​TTC​AGT​CCT​GTC​CAT-3′; **hs-IL-6**: forward 5′- AAA​GAT​GGC​TGA​AAA​AGA​TGG​ATG​C-3′, reverse 5′-ACA​GCT​CTG​GCT​TGT​TCC​TCA​CTA​C-3′; **hs-Ptgs2**: forward 5′-TCC​CTT​GGG​TGT​CAA​AGG​TAA​A-3′, reverse 5′-TGG​CCC​TCG​CTT​ATG​ATC​TG-3′.

### Bacterial Burden Determination

Here, 1 × 10^5^ MDMs were plated in a 96-well tissue culture plate and infected with Mtb HN878 at MOI- 2 as described above. Cells were then treated with 100 nM triptolide for up to 24- and 48-h postinfection. At 4, 24, and 48 h postinfection, cells were lysed in Triton X-100, and serial dilutions were plated on Middlebrook 7H10 agar plates and incubated for 15 days at 37 °C in 5% CO_2_. Colony-forming units (CFUs) for each sample were enumerated to determine bacterial burden.

### Microscopy

MDMs were treated as above and before harvesting for total RNA extraction, MDMs were observed with the ZOE Fluorescent Cell Imager using the bright-field light. Pictures were taken at 2x magnificence.

### alamarBlue Assay

2 × 10^^5^ Mtb HN878 or Mtb H37rv CFU were seeded on 96-well plates in 50 ul Mtb growth medium (7H9 media supplemented with OADC and glycerol). Then 50 ul of growth medium containing 16–2000 nM triptolide alone, rifampicin alone (positive control; known to kill extracellular Mtb), or mixtures of both (1:1 v/v ratio) was added on top of the cells to make up a final drug concentration of 8–1000 nM. DMSO was used as a vector control. 10 ul alamarBlue ^R^ reagent (DAL1025, Thermo Fisher, United States) was added to the plate on day 14, and the plate was incubated for 4 h at 37°C in the dark. Mtb growth was monitored by absorbance (OD) reading at 570 nm.

### Graphs and Statistical Analysis

Results were plotted using GraphPad Prism v8 and analyzed using an unpaired, two-tailed *t*-test or two-way ANOVA as relevant, with *p* values represented as *p* > 0.05 ns, *p* < 0.05*, *p* < 0.01**, and *p* < 0.001***.

## Results

### Triptolide Treatment Increased the Expression of lincRNA-p21 but Decreased the Expression of lncRNA-PACER in Monocyte-Derived Macrophages

**FIGURE 1:**
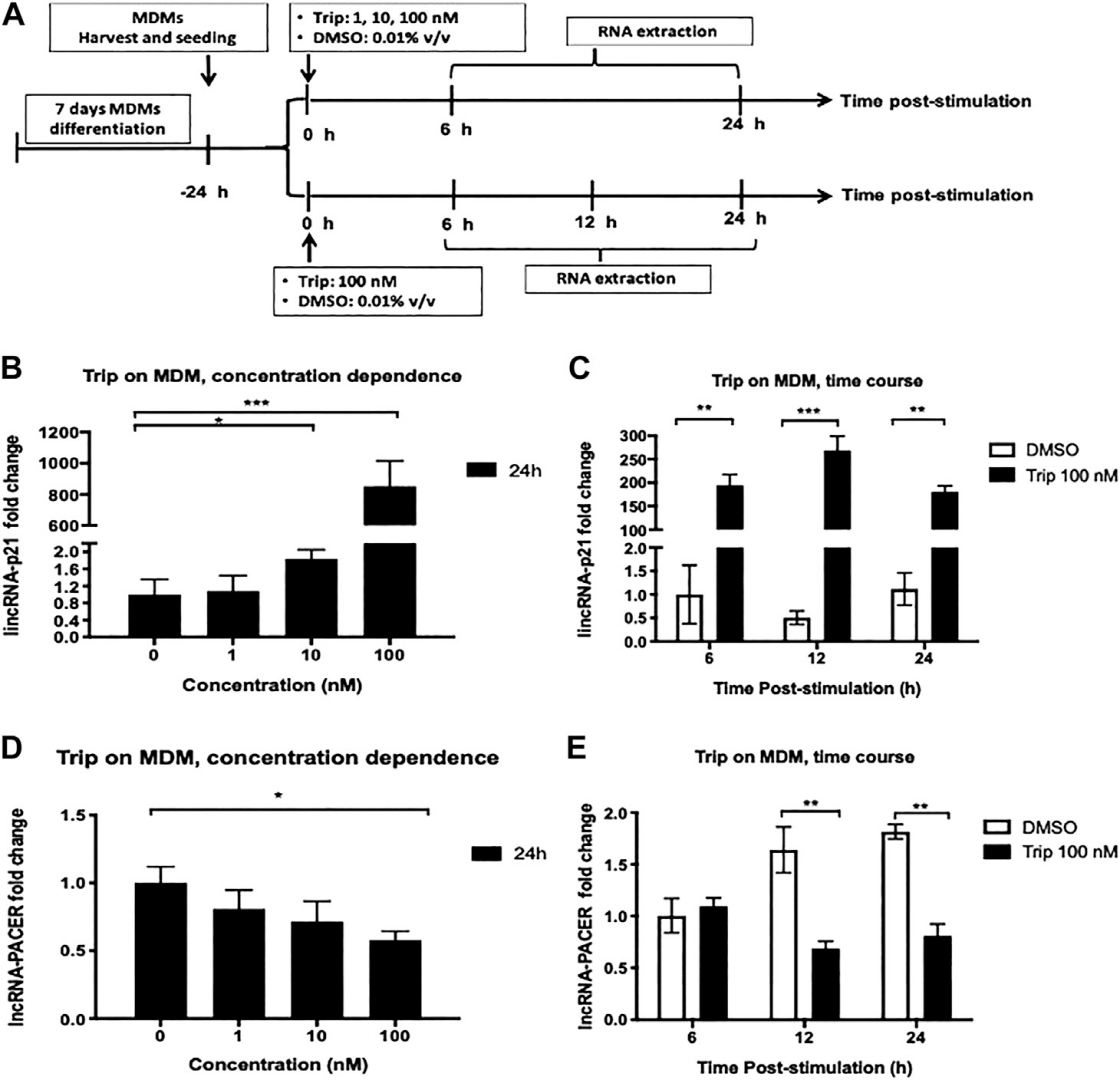
Triptolide treatment modulates the expression of lincRNA-p21 and lncRNA PACER in resting monocyte-derived macrophages (MDMs). **(A)**: Graphical summary for concentration dependence and time dependence experiments. Concentration dependence **(B, D)**: MDMs were treated with increasing concentrations of triptolide for 6 and 24 h. DMSO was used as a vector control. Total RNA was extracted, and then lincRNA-p21 **(B)** and lncRNA-PACER **(D)** expression levels were assessed by RT-qPCR. Time dependence **(C, E)**: MDMs were treated with 100 nM triptolide for 6, 12, and 24 h. DMSO was used as a vector control. Total RNA was extracted, and then lincRNA-p21 **(C)** and lncRNA-PACER **(E)** expression levels were assessed by RT-qPCR. Relative expression was calculated using the −ΔΔCt method. *p < 0.05; **p < 0.01; ***p < 0.001.

We investigated whether triptolide (Trip) modulates the expression of lincRNA-p21 and lncRNA-PACER in monocyte-derived macrophages (MDMs). MDMs were treated with increasing concentrations of Trip for 24 h or with 100 nM Trip for 6, 12, and 24 h (Figure 1A). Total RNA was then extracted and lncRNAs expression levels were assessed by RT-qPCR. We found that Trip treatment induced a significant increase of the expression of lincRNA-p21 (853 ± 163 folds with 100 nM Trip) but inhibited the expression of lncRNA-PACER in MDMs in a concentration (Figures 1B,D) and time-dependent manner (Figures 1C,E).

MDMs were also treated with increasing concentrations of Trip for 6 h. We still observed a concentration-dependent increase of lincRNA-p21 expression (Supplementary Figure S1A), but no effect on lncRNA-PACER expression (Supplementary Figure S1B). This indicated that lincRNA-p21 upregulation is an early event whereas lncRNA-PACER downregulation is a late event in MDMs response to Trip.

### High-Concentration Triptolide Treatment Increased the Expression of Pro-Inflammatory Genes Ptgs-2 and IL-6

**FIGURE 2:**
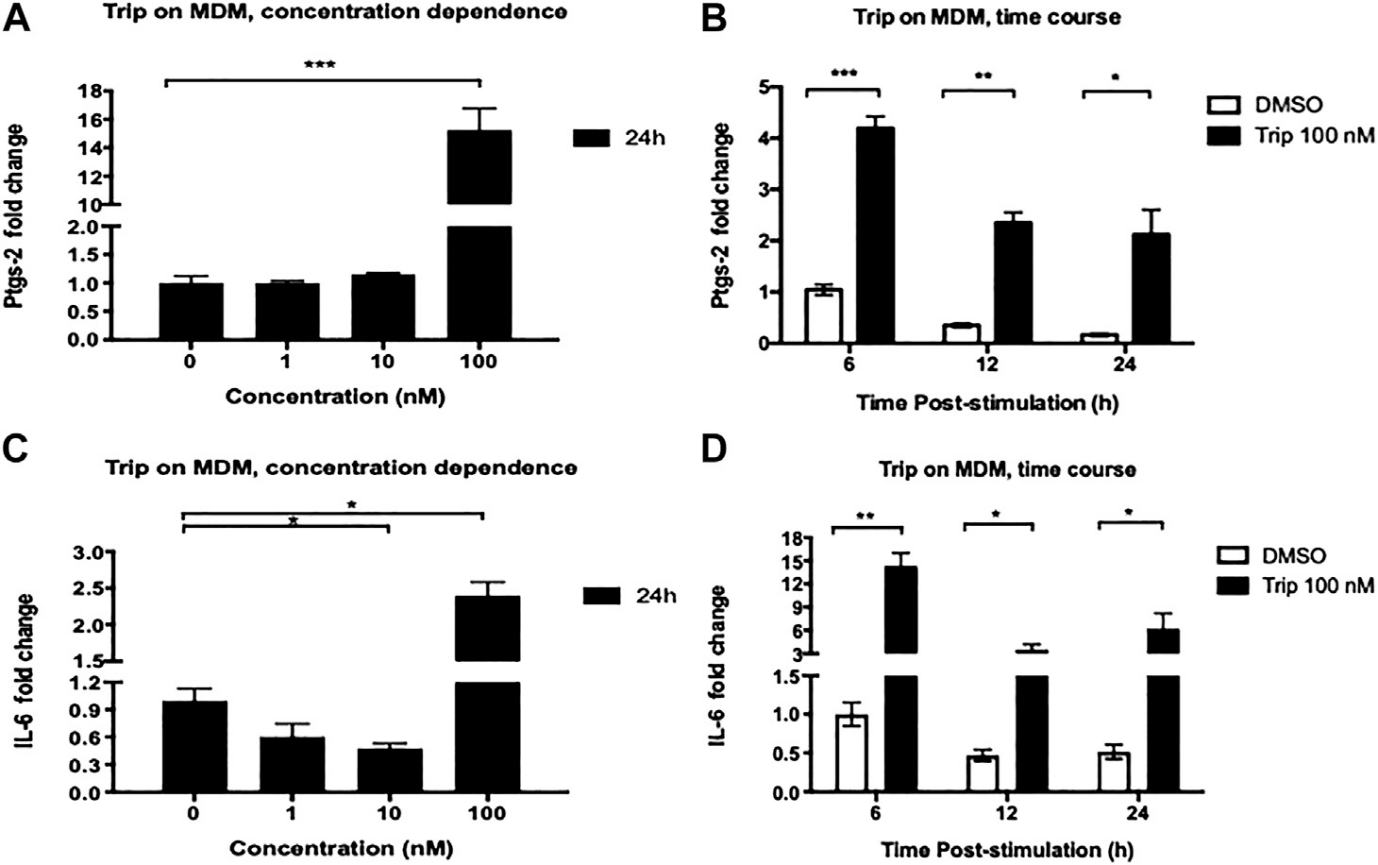
Triptolide treatment modulates the expression of lncRNA-associated pro-inflammatory genes Ptgs-2 and IL-6 in resting monocyte-derived macrophages (MDMs). Concentration dependence **(A, C)**: MDMs were treated with increasing concentrations of triptolide for 6 and 24 h. DMSO was used as a vector control. Total RNA was extracted, and then Ptgs-2 **(A)** and IL-6 **(C)** expression levels were assessed by RT-qPCR. Time dependence **(B, D)**: MDMs were treated with 100 nM triptolide for 6, 12, and 24 h. DMSO was used as a vector control. Total RNA was extracted, and then Ptgs-2 **(B)** and IL-6 **(D)** expression levels were assessed by RT-qPCR. Relative expression was calculated using the −ΔΔCt method. *p < 0.05; **p < 0.01; ***p < 0.001.

lncRNA-PACER has been reported as a positive regulator of its proximal pro-inflammatory gene Ptgs-2 in several cell types and conditions. We then sought to assess the effect of Trip on the expression of Ptgs-2 and associate it with lncRNA-PACER expression. MDMs were treated as above and we found that like with lncRNA-PACER, MDMs treatment with low-concentration Trip (1, 10 nM) for 24 h did not significantly affect Ptgs-2 expression (Figure 2A). However, contrary to lncRNA-PACER, high-concentration (100 nM) Trip treatment significantly increased Ptgs-2 expression in a time-dependent manner, with up to 15-fold increase in Ptgs-2 mRNA level at 24 h posttreatment (Figures 2A,B); thus, indicating an inverse relation between Trip-induced expression of lncRNA-PACER and its proximal gene Ptgs-2 in MDMs. We also assessed the effect of Trip on the expression of IL-6 which is a lincRNA-p21’s target. MDMs treatment with low-concentration Trip led to significant decrease of IL-6 mRNA level (Figure 1C). However, high-concentration Trip significantly increased IL-6 mRNA level in a time-dependent manner, reaching up to 15-fold increase at 6 h posttreatment (Figure 1D).

MDMs were also treated with increasing concentrations of Trip for shorter time (6 h). We still observed the same Ptgs-2 expression pattern as with the 24 h treatment (Supplementary Figure S1C), but we noticed a significant downregulation of IL-6 expression by 100 nM Trip, which was contrary to the upregulation observed at 24 h treatment (Supplementary Figure S1D). This indicates that 100 nM Trip treatment triggers an early induction of Ptgs-2 and inhibition of IL-6 expression in MDMs. However, prolonged stimulation with Trip leads to increased IL-6 expression at late time point in resting MDMs.

### Triptolide Pretreatment Further Increases Mtb HN878-Induced Expression of lincRNA-p21 and lncRNA-PACER

Since Trip modulated the expression of lincRNA-p21 and lncRNA-PACER in resting MDMs, we then sought whether it also modulates their expression in *Mycobacterium tuberculosis* (Mtb)-induced inflammation model.

**FIGURE 3:**
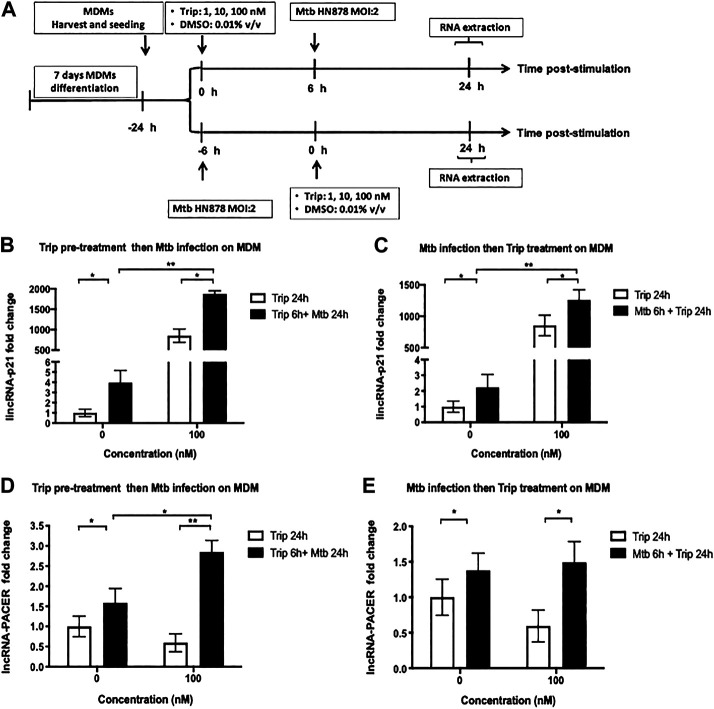
Triptolide pretreatment or posttreatment further increases Mtb HN878-induced expression of lincRNA-P21 and lncRNA-PACER in MDMs. **(A)**: Graphical summary for the experiments on Mtb HN878 infection of MDMs pre- and post-triptolide treatments. Triptolide pretreatment **(B, D)**: MDMs were pretreated with the indicated concentrations of triptolide for 6 h then infected with Mtb HN878 MOI:2 up to 24 h. DMSO was used as a vector control. Total RNA was extracted and then lincRNA-p21 **(B)** and lncRNA-PACER **(D)** expression levels were assessed by RT-qPCR. Triptolide post-treatment **(C, E)**: MDMs were infected with Mtb HN878 MOI:2 for 6 h then treated with the indicated concentrations of triptolide up to 24 h. DMSO was used as a vector control. Total RNA was extracted, and then lincRNA p-21 **(C)** and lncRNA-PACER **(E)** expression levels were assessed by RT-qPCR. Relative expression was calculated using the −ΔΔCt method. *p < 0.05; **p < 0.01; ***p < 0.001.

MDMs were pretreated with 100nM Trip or DMSO (vector control) for 6 h, then infected with the hypervirulent Mtb HN878 clinical strain (MOI:2) for up to 24 h. For the posttreatment experiment, MDMs were infected with Mtb HN878 (MOI:2) for 6 h, then treated with 100 nM Trip or DMSO for up to 24 h (Figure 3A).

Mtb infection led to significant induction of lincRNA-p21 and lncRNA-PACER in MDMs (up to 4- and 1.5-fold increase for lincRNA-p21 and lncRNA-PACER, respectively) (Figure 3).

Treatment with 100 nM Trip 6 h before Mtb-infection led to further increase of Mtb-induced expression of these lncRNAs with more than 400-fold lincRNA-p21 (Figure 3B) and two-fold lncRNA-PACER (Figure 3D) induction in Trip-pretreated and Mtb-infected as compared to DMSO-pretreated and Mtb-infected MDMs.

Treatment with 100 nM Trip 6 h after Mtb infection further increased Mtb-induced expression of lincRNA-p21 (more than 600-fold) when compared to DMSO-pretreated and Mtb-infected MDMs (Figure 3C). Posttreatment with 100 nM Trip; however, it does not further increase Mtb-induced expression of lncRNA-PACER (Figure 3E).

Low-concentration Trip pretreatment or posttreatment further increased Mtb-induced expression of lincRNA-p21 and lncRNA-PACER in MDMs (Supplementary Figure S1).

These results indicate a synergistic effect of Trip treatment and Mtb infection on the expression of lincRNA-p21, but rather antagonistic effect on the expression of lncRNA-PACER.

### Triptolide Pre- and Posttreatment Affect Mtb HN878-Induced Expression of Ptgs-2 and IL-6

**FIGURE 4:**
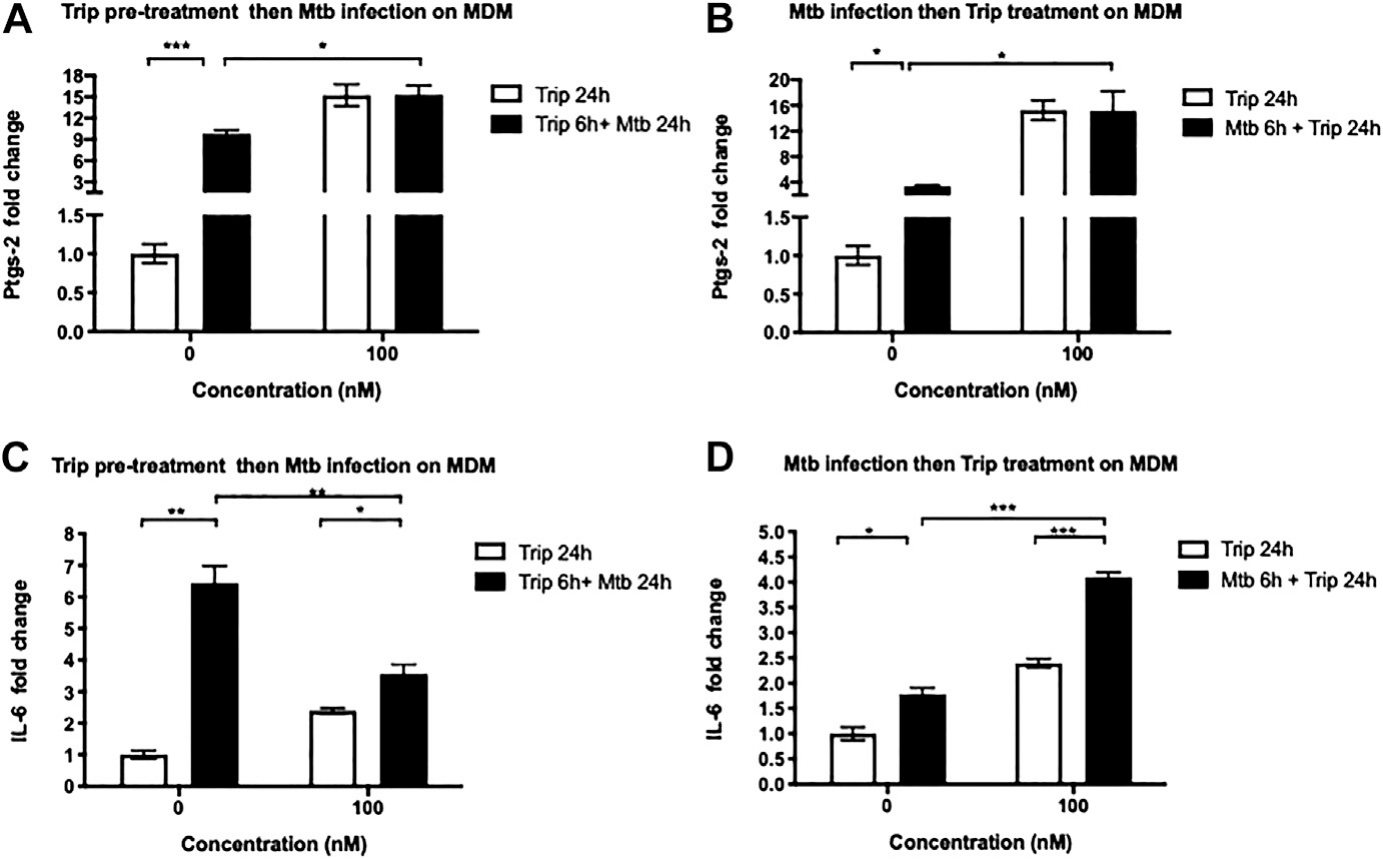
Triptolide pretreatment decreases Mtb HN878-induced expression of IL-6 and Ptgs-2 but posttreatment further increases their expression in MDMs. triptolide pretreatment **(A, C)**: MDMs were pretreated with the indicated concentrations of triptolide for 6 h then infected with Mtb HN878 MOI:2 up to 24 h. DMSO was used as vector control. Total RNA was extracted then IL-6 **(A)** and Ptgs-2 **(C)** expression levels were assessed by RT-qPCR. Triptolide post-treatment **(B, D)**: MDMs were infected with Mtb HN878 MOI:2 for 6 h then treated with the indicated concentrations of triptolide up to 24 h. DMSO was used as vector control. Total RNA was extracted then IL-6 **(B)** and Ptgs-2 **(D)** expression levels were assessed by RT-qPCR. Relative expression was calculated using the −ΔΔCt method. *p < 0.05; **p < 0.01; ***p < 0.001.

We further tested whether Trip also modulates the expression of these lncRNAs’ targets in an Mtb-induced inflammation model. MDMs were treated as above, and we found that Mtb infection significantly induced the expression of Ptgs-2 and IL-6 (up to 9- and 6.5-fold increase for Ptgs-2 and IL-6, respectively) (Figure 4).

Treatment with 100 nM Trip 6 h before (Figure 4A) or after (Figure 4B) Mtb infection further increased Mtb-induced expression of Ptgs-2 in Trip-treated and Mtb-infected than that in DMSO-treated and Mtb-infected MDMs (1.5- and 4-fold increase in Trip pretreatment and posttreatment, respectively). We noticed, however, no difference in Ptgs-2 expression between 100 nM Trip-treated uninfected and 100 nM Trip-treated Mtb-infected MDMs (Figures 4A,B), which indicates that 100 nM Trip treatment is the main driver of Ptgs-2 induction, which is not further enhanced by Mtb infection.

We also observed that pretreatment with low-concentration Trip inhibited Mtb-induced expression of Ptgs-2 (Supplementary Figure S3A), whereas posttreatment further heightened Mtb-induced expression of Ptgs-2 in MDMs (Supplementary Figure S3C). This highlights a clear difference between the effects of low-concentration Trip pretreatment and posttreatment on Mtb-induced expression of Ptgs-2.

Treatment with 100 nM Trip 6 h before Mtb infection led to a significant 2-fold decrease of Mtb-induced expression of IL-6 in Trip-treated Mtb-infected as compared to DMSO-treated Mtb-infected MDMs (Figure 4C). Conversely, treatment with 100 nM Trip 6 h after Mtb infection led to a significant two-fold increase of Mtb-induced IL-6 expression in Trip-treated Mtb-infected as compared to DMSO-treated Mtb-infected MDMs (Figure 4D). This indicates an antagonistic effect of 100 nM Trip pretreatment on Mtb-induced upregulation of IL-6, but a synergistic effect of 100 nM Trip posttreatment on Mtb-induced upregulation of IL-6.

Similar to treatment with 100 nM Trip, pretreatment with low-concentration Trip inhibited Mtb-induced expression of IL-6 (Supplementary Figure S3B), whereas posttreatment further heightened Mtb-induced expression of IL-6 in MDMs (Supplementary Figure S3D).

### Triptolide Treatment Decreases Mtb HN878 Growth in Monocyte-Derived Macrophages but Not in Culture Medium

**FIGURE 5:**
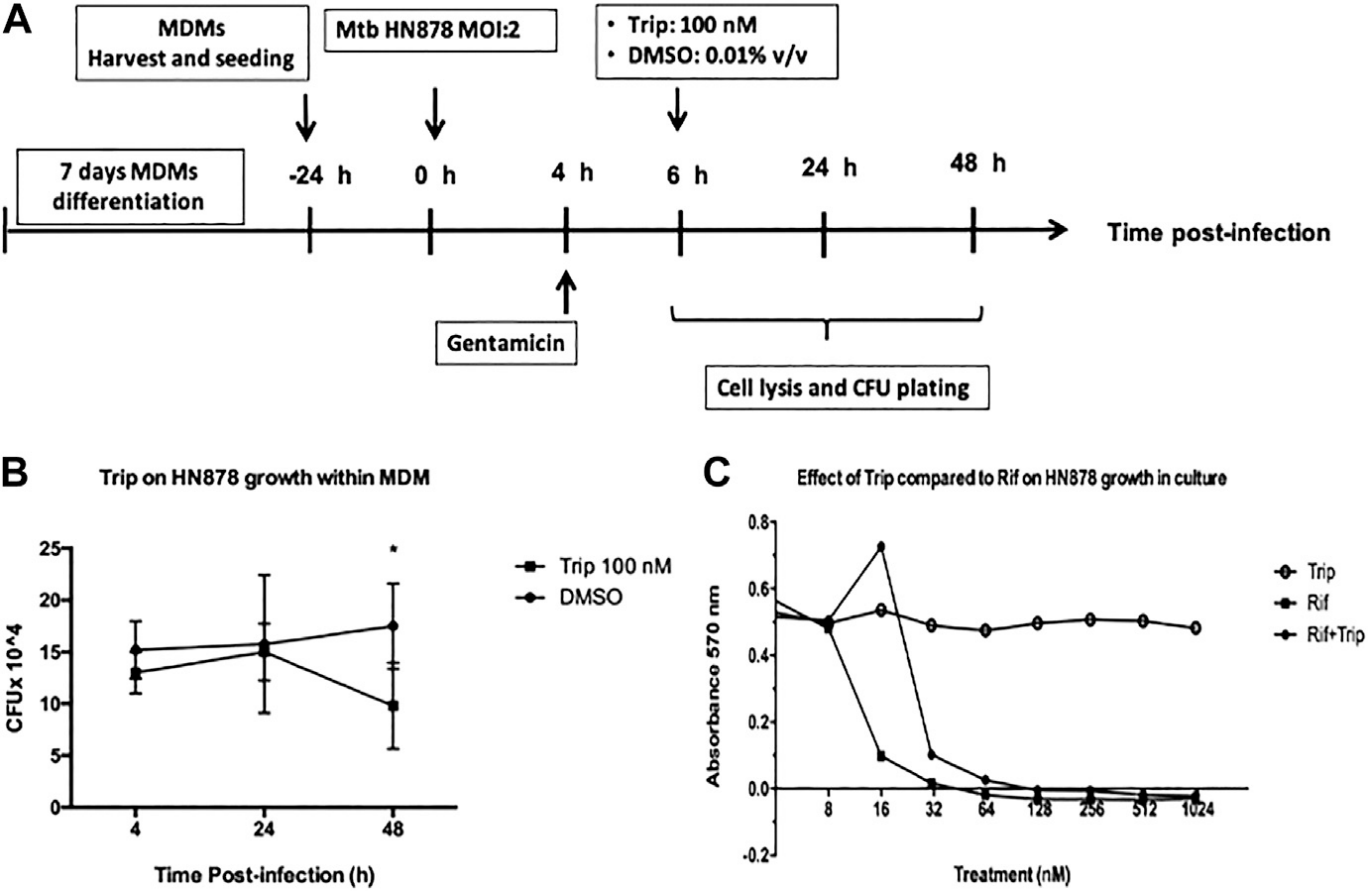
Triptolide treatment decreases Mtb HN878 growth in MDMs but not in the culture medium. **(A)**: Graphical summary for the experiment on the effect of triptolide on Mtb HN878 growth within MDMs. Triptolide reduces Mtb HN878 CFU in MDMs **(B)**: 10^5 MDMs were infected with Mtb HN878 MOI:2 for 4 h then treated with 100 nM triptolide for 24 and 48 h. DMSO was used as vector control. At each time point, BMDMs were lysed and the lysate plated for determination of CFU 15 days later. Triptolide does not affect Mtb HN878 growth in culture medium **(C)**: 2 × 10^5 Mtb HN878 CFU was seeded on 96-well plates and then treated with triptolide, rifampicin, or mixtures of both at different concentrations. DMSO was used as a vector control. alamarBlue stain was added to the plate on day 14 and OD was read at 570 nm *p < 0.05; **p < 0.01; ***p < 0.001.

We then sought to investigate how Trip-induced changes in lncRNA-PACER and lincRNA-p21 expression associates with Mtb faith within infected MDMs. MDMs were infected with Mtb HN878 for 6 h, and then treated with 100 nM Trip for 24 and 48 h (Figure 5A). Using the CFU count method, we found that 100 nM Trip treatment significantly decreased the MDMs intra-cellular growth of Mtb HN878 at 48 h postinfection (Figure 5B). Mtb HN878 infection induced MDM cell death when compared to uninfected MDMs, and Trip treatment further increased MDMs cell death in a dose-dependent manner (Supplementary Figure S4).

To check whether Trip acts by direct targeting of Mtb HN878, the bacterium was seeded on 96-well plates and then treated with Trip, rifampicin (known to kill Mtb), or mixtures of both at different concentrations for 14 days. alamarBlue cell viability assay showed that Trip does not affect Mtb HN878 growth through direct interaction between Trip and Mtb HN878 (Figure 5C). Moreover, Trip also did not affect the growth of the virulent Mtb H37Rv strain through direct interaction (Supplementary Figure S5). Together, these show that decreased Mtb HN878 growth in Trip-treated MDMs was not as a result of direct targeting of Mtb HN878 by Trip, but rather evoking MDMs intracellular killing mechanisms.

## Discussion

We report for the first time that triptolide induces a significant upregulation of lincRNA-p21 within resting MDMs in a time- and concentration-dependent manner. Indeed, lincRNA-p21 plays a crucial role in stress and DNA damage response through the induction of p53-dependent cell cycle arrest and apoptosis in numerous cell types (Huarte et al., 2010; Dimitrova et al., 2014; Tu et al., 2017; Jin et al., 2019). On the other hand, triptolide is a cytotoxic compound that induces DNA damage, cell cycle arrest, apoptosis, and autophagy in several cell types (Park and Kim, 2013; Zhang et al., 2016; You et al., 2018). Our results are in line with previous reports that indicated increased lincRNA-p21 expression in several cells and tissues exposed to genotoxic agents (Recio et al., 2013; Gezer et al., 2014; Tang et al., 2015). This urges for an in-depth investigation of the role of lincRNA-p21 as an early mediator of triptolide’s cytotoxic effect on MDMs.

Besides its cytotoxicity, triptolide possesses anti-inflammatory activities, which it performs *via* downregulation of NF-kb– and AP-1–controlled pro-inflammatory molecules such as TNF-α, IL-6, IL-12, and Ptgs-2 in macrophages and dendritic cells (Liu et al., 2007; Lu et al., 2014). lincRNA-p21 may also mediate the anti-inflammatory activities of triptolide in resting MDMs. In support of this hypothesis, it was shown that lincRNA-p21 is induced by p53 and mediates the anti-inflammatory activities of this transcription factor (Komarova et al., 2005; Huarte et al., 2010). Moreover, it was reported that methotrexate, a drug with anti-inflammatory properties used for the treatment of rheumatoid arthritis, induced the expression of lincRNA-p21, which in turn inhibited the activity of the pro-inflammatory transcription factor NF-kB in THP-1 monocyte cell lines and PBMCs (Pahl, 1999; Spurlock et al., 2014). Our findings highlight, also for the first time, that triptolide inhibits the expression of the pro-inflammatory lncRNA-PACER (Krawczyk and Emerson, 2014). Although lncRNA-PACER downregulation was noticeable at a late exposure time (24 h) as opposed to lincRNA-p21 induction which was triggered early (6 h), these concordant findings point to these lncRNAs as mediators of triptolide’s cytotoxic and anti-inflammatory activities in resting MDMs.

To further support this conjecture, we explored how triptolide-induced alteration of lincRNA-p21 and lncRNA-PACER expression associated with the expression of some of their target genes.

Pro-inflammatory cytokine IL-6 was described as a target of lincRNA-p21 in LPS-stimulated BV2 microglia cells (Ye et al., 2018a). We found that contrary to lincRNA-p21, low-concentration triptolide led to significant downregulation of IL-6 in resting MDMs, which is concordant with the conception that lincRNA-p21 mediate at least partially the anti-inflammatory activity of triptolide, given that other mechanisms, such as the inhibition of NF-kB signaling pathway are also involved in the downregulation of IL-6 by triptolide (Wang et al., 2014; Chen et al., 2018). Unexpectedly, we found, however, that high concentration of triptolide significantly upregulated IL-6 expression. Although the reason for this is not known, it is worth mentioning that triptolide displays dose-dependent effects on gene expression and faith of several cell types (Kiviharju et al., 2002; Tamgue and Lei, 2017). Also, IL-6 is a pleiotropic cytokine playing controversial role in inflammation and cell faith which seem to be context- and tissue-dependent (Mauer et al., 2015; Linnemann et al., 2017). Last, lincRNA-p21 showed anti-inflammatory or pro-inflammatory roles depending on the stimulators, cell types, or specific disease context (Spurlock et al., 2014; Yang et al., 2014; Zhou et al., 2016; Ye et al., 2018a).

lncRNA-PACER has been described as a positive regulator of its proximal pro-inflammatory gene Ptgs-2 (Cox-2) *via* mechanisms involving the sequestration of repressive NF-κB subunit p50 away from Ptgs-2 promoter (Krawczyk and Emerson, 2014). Contrary to lncRNA-PACER, low-concentration triptolide did not affect Ptgs-2 expression, whereas high concentration significantly induced Ptgs-2 expression in MDMs, possibly as an attempt by the cell to repair its plasma membrane disrupted by high-concentration cytotoxic triptolide. It has indeed been shown that Ptgs-2 drives the biosynthesis of the prostanoid PGE2, a lipid mediator involved in the repair of disrupted plasma membrane (Abebe et al., 2011). Our findings suggest that either triptolide mainly regulates the expression of Ptgs-2 in resting MDMs through lncRNA-PACER–independent mechanisms as already reported (Chun and Surh, 2004; De Moraes et al., 2007), or that lncRNA-PACER acts as a negative regulator of Ptgs-2 in resting MDMs and similar to the action of lincRNA-cox-2. This is another known regulator of Ptgs-2, which mediates both activation and repression of several immune response genes (Carpenter et al., 2013).

Although lincRNA-p21 was induced early and lncRNA-PACER lately following triptolide treatment, their target genes IL-6 and Ptgs-2 were both dysregulated at early time points, suggesting the involvement of additional regulatory mechanisms. Indeed, several mechanisms were involved in the tight control of pro-inflammatory gene expression, which will keep a balanced expression level and prevent cell-detrimental excessive inflammation. Ptgs-2 expression is thus regulated through p53 and NF-kB pathways (Chun and Surh, 2004; De Moraes et al., 2007), meanwhile IL-6 expression is controlled through NF-kB signaling pathway and triptolide triggers early downregulation of IL-6 expression *via* NF-kB signaling pathway (Wang et al., 2014; Chen et al., 2018). lincRNA-p21 and lncRNA-PACER gain-/loss-of-function experiments are warranted to unequivocally identify their targets in MDMs and to highlight their precise contribution to triptolide’s biological activities on resting MDMs.

Since lincRNA-p21 and lncRNA-PACER were mainly dysregulated in inflammatory conditions, we were then interested in finding whether these lncRNAs mediate triptolide’s activities in an inflammatory setting as well. We opted for a *Mycobacterium tuberculosis* (Mtb) infection model, which is known to induce an inflammatory response and to trigger cell death of infected macrophages through mechanisms that are not fully understood. Also, the molecular mechanisms underlying the effect of triptolide on the effector functions of MDMs have not been investigated before. lincRNA-p21 was highly induced in MDMs upon infection with the hypervirulent clinical Mtb HN878 strain. This induction was further exacerbated in MDMs exposed to triptolide before or after the infection. This indicates triptolide and Mtb infection trigger synergistic pathways leading to heightened expression of lincRNA-p21. lincRNA-p21’s target gene IL-6 was downregulated in MDMs exposed to triptolide before Mtb infection, suggesting that like in resting MDMs, lincRNA-p21 may also mediate IL-6 targeting by triptolide in inflammatory settings. We observed, however, that IL-6 was upregulated in MDMs exposed to triptolide after Mtb infection. The reasons for this need further investigation. lncRNA-PACER was slightly induced (two-fold) in MDMs upon infection with the hypervirulent clinical Mtb HN878 strain. This induction was further exacerbated in MDMs exposed to high-concentration triptolide before but not after Mtb infection. lncRNA-PACER’s target gene Ptgs-2 was also upregulated upon Mtb infection, which was concordant with previous reports of its induction by the Mtb H37Rv laboratory strain (Xiong et al., 2018). We found, however, that Ptgs-2 was induced to a lesser magnitude when compared to samples treated with high-concentration triptolide alone without Mtb infection. Consequently, triptolide-induced Ptgs-2 expression was not further enhanced by Mtb infection. This indicates that high-concentration triptolide induces the strongest upregulation of Ptgs-2, thus masking any further effect caused by Mtb infection. This was substantiated by the observation that low-concentration triptolide pretreatment inhibited Mtb-induced expression of Ptgs-2, but posttreatment further increased Mtb-induced expression of Ptgs-2. Hence, this result again suggests lncRNA-PACER acts as a negative regulator of Ptgs-2 in Mtb-infected MDMs or that triptolide mainly regulates the expression of Ptgs-2 through lncRNA-PACER–independent mechanisms.

Triptolide-induced Ptgs-2 expression may promote the repair of disrupted plasma membrane which is known to prevent Mtb-beneficial necrotic cell death and mycobacterial escape (Abebe et al., 2011) and to promote Mtb-detrimental apoptotic death of host MDMs (Divangahi et al., 2009). Concordantly, the downregulation of Ptgs-2 in MDMs treated with low-concentration triptolide may indicate Mtb actions to escape the immune response (Chen et al., 2008). These conjectures need further investigation.

Last, we observed that triptolide induced the intracellular killing of Mtb HN878 in MDMs. We thus highlight an association between heightened expression of lincRNA-p21, lncRNA-PACER, and Mtb HN878 growth inhibition in triptolide-treated MDMs. We speculate that the killing mechanism may involve lncRNA-PACER– and/or lincRNA-p21–mediated enhancement of MDMs effector killing functions. Indeed, it is known that Mtb hijacks host non-coding RNAs to evade the immune response and favor its persistence within the infected host (Wang et al., 2015; Huang et al., 2018; Roy et al., 2018; Tamgue et al., 2019). lincRNA-p21 is a positive regulator of apoptosis, a programmed cell death that contributes to host defense against intracellular pathogens such as Mtb (Lam et al., 2017). It was shown that HIV-1 survives in infected macrophages by inhibiting apoptosis through induced degradation of lincRNA-p21 (Barichievy et al., 2018). The lncRNA-PACER promotes the activation of macrophages toward the pro-inflammatory M1 phenotype known to be very efficient in killing Mtb (Ye et al., 2018b). lincRNA-p21 and lncRNA-PACER target genes IL-6 and Ptgs-2 are pro-inflammatory genes playing key role in cellular immune response against Mtb (Nagabhushanam et al., 2003; Jung et al., 2017; Xiong et al., 2018). Indeed, the mRNA levels of these effector molecules were highly increased in Mtb-infected MDMs which were treated with triptolide 100 nM than those treated with DMSO. IL-6 is a pro-inflammatory cytokine that promotes classical activation of macrophage and intracellular killing of Mtb (Martinez et al., 2013; Jung et al., 2017). Ptgs-2 promotes apoptosis (Divangahi et al., 2009) and autophagy (Xiong et al., 2018) of Mtb-infected macrophages, thus accelerating the killing and elimination of Mtb within the host. The heightened expression of these lncRNAs and their targets IL-6 and Ptgs-2 may explain the higher Mtb killing within the triptolide-treated MDMs than that in DMSO-treated MDMs.

In conclusion, this study provided first evidences that triptolide modulates the expression of lncRNA-PACER and lincRNA-p21 as well as of their target genes IL-6 and Ptgs-2 in both resting and Mtb infected human macrophages. Further, triptolide inhibited Mtb growth within MDMs (Supplementary Figure S6). Based on these results and available literature, we were only able to conjecture a link between these lncRNAs expression, the specific enhancement of pro-inflammatory activities in macrophages and the intracellular Mtb-killing activities of triptolide, which is a limitation to the current study. Therefore, our pathfinding work prompts for further research, where the usage of lincRNA-p21 and/or lncRNA-PACER gain-/loss-of-function systems (*in vitro* lncRNAs gene knockdown and gene-deficient mouse model) will unequivocally identify or confirm these lncRNAs’ target genes in MDMs and uncover the precise mechanisms by which they mediate triptolide’s biological activities (including balancing of MDMs activation level/state, inhibition of Mtb growth, and enhancement of MDMs autophagy, apoptosis, phagocytic activity, and other effector killing activities).

## Data Availability

The raw data supporting the conclusion of this article will be made available by the authors, without undue reservation, to any qualified researcher.
