# Supplementary figures and images for: Triptolide Modulates the Expression of Inflammation-Associated lncRNA-PACER and lincRNA-p21 in Mycobacterium tuberculosis–Infected Monocyte-Derived Macrophages
Source: Front Pharmacol. 2021 Apr 12;12:618462. doi: 10.3389/fphar.2021.618462 (PMC8071990; doi:10.3389/fphar.2021.618462)

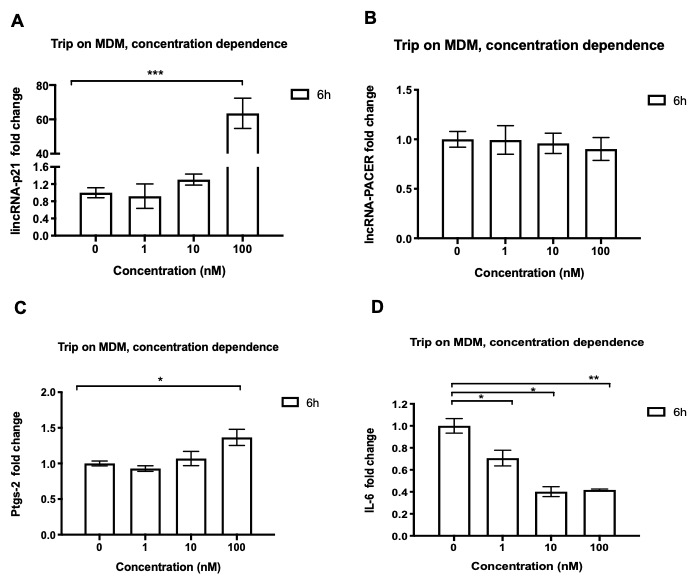

Supplement: Supplementary file 1 [file image1.jpeg]

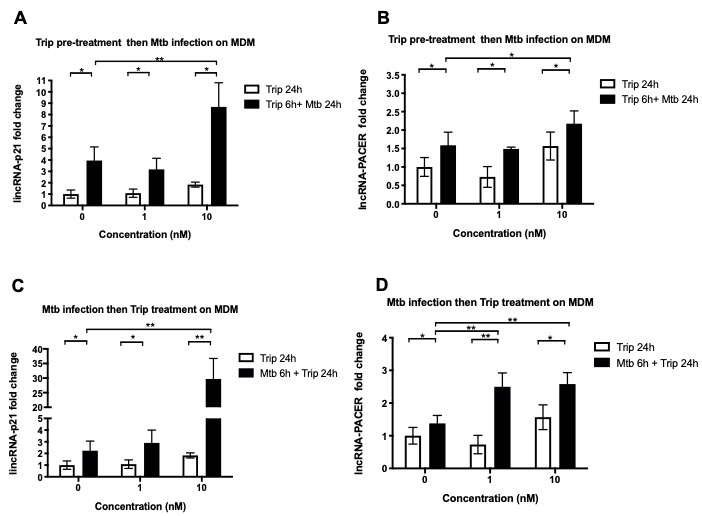

Supplement: Supplementary file 2 [file image2.jpeg]

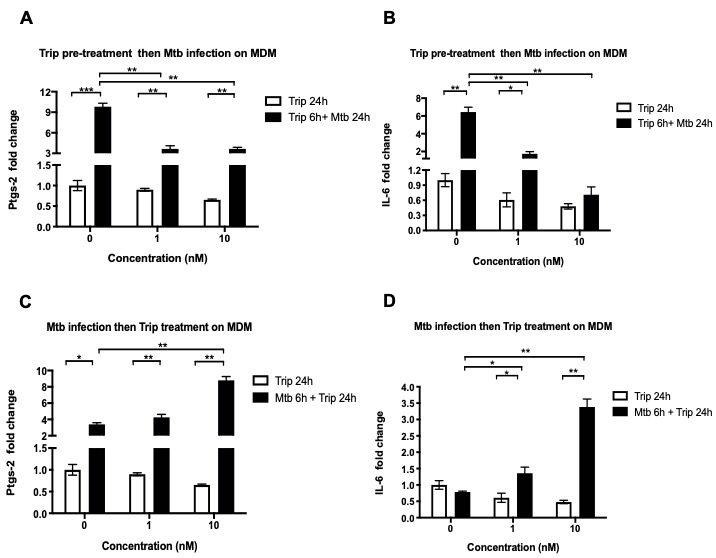

Supplement: Supplementary file 3 [file image3.jpeg]

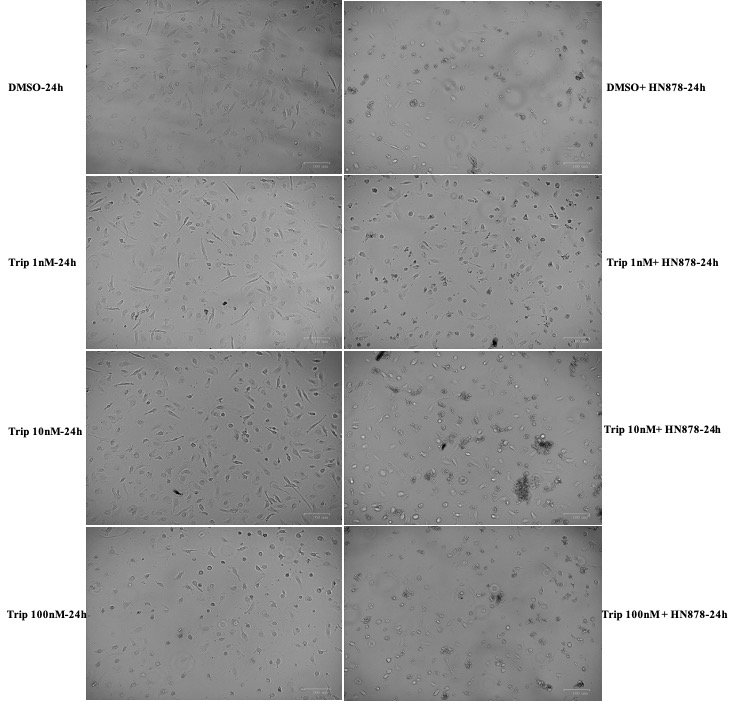

Supplement: Supplementary file 4 [file image4.jpeg]

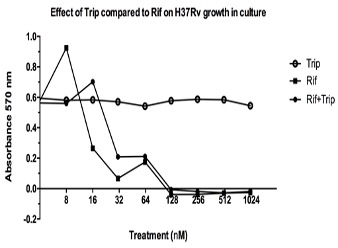

Supplement: Supplementary file 5 [file image5.jpeg]

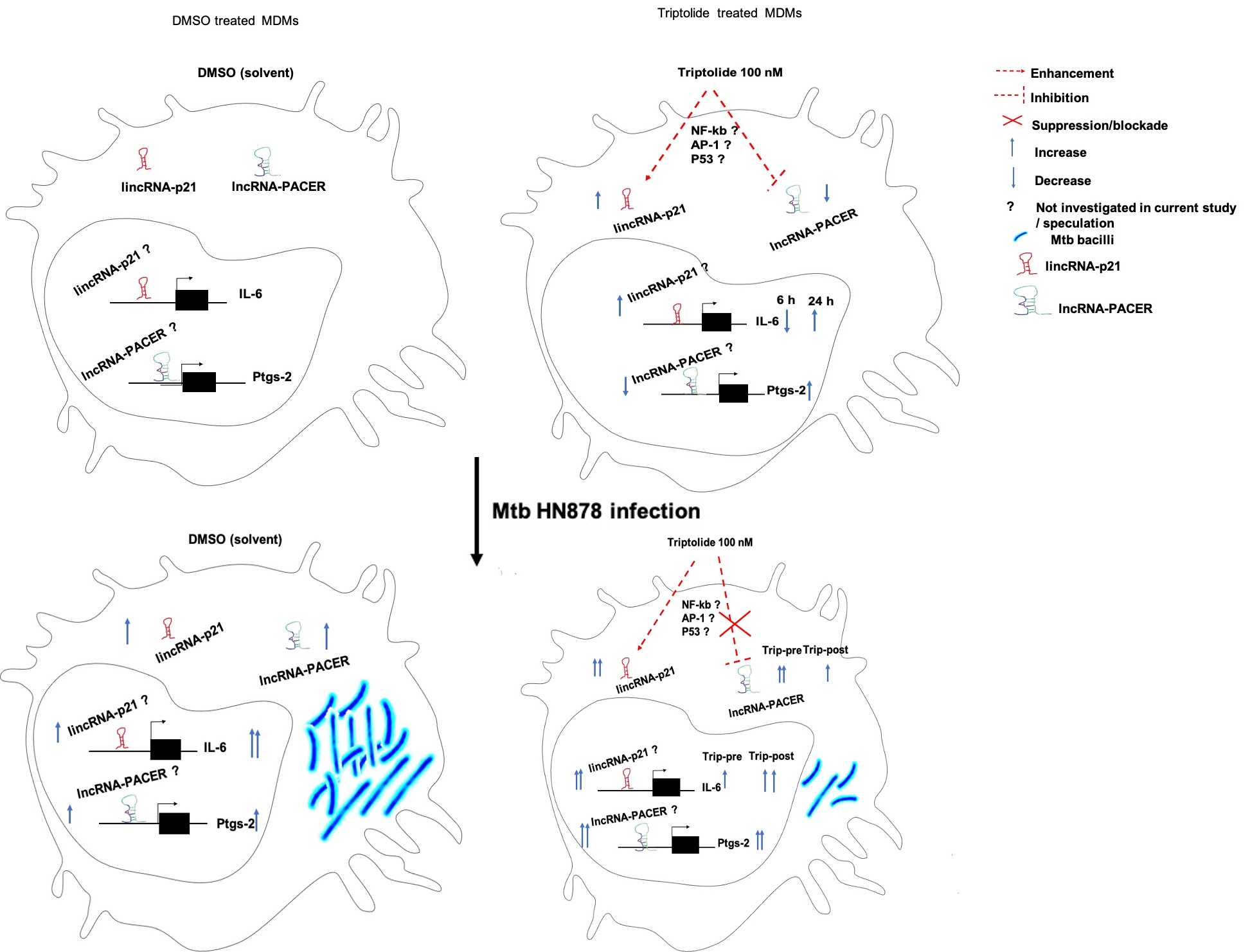

Supplement: Supplementary file 6 [file image6.jpeg]
